# How is equity captured for colorectal, breast and cervical cancer incidence and screening in the Republic of Ireland: A review

**DOI:** 10.1016/j.pmedr.2023.102405

**Published:** 2023-09-17

**Authors:** Sophie Mulcahy Symmons, Paul Leavy, Laura Heavey, Caroline Mason Mohan, Amanda Drury, Aoife De Brún

**Affiliations:** a School of Nursing, Midwifery and Health Systems, University College Dublin, Ireland; b UCD Centre for Interdisciplinary Research, Education, and Innovation in Health Systems (UCD IRIS), School of Nursing, Midwifery and Health Systems, University College Dublin, Ireland; c Centre for Health Policy and Management, School of Medicine, Trinty College Dublin, Ireland; d Department of Public Health Medicine, National Screening Service, King’s Inn House, 200 Parnell Street, Dublin 1, Ireland; e School of Nursing, Psychotherapy and Community Health, Dublin City University, Glasnevin, Dublin 9, Ireland

**Keywords:** Early detection of cancer, Cancer screening, Incidence, Cervical cancer, Breast cancer, Colorectal cancer, Health inequities, Health inequalities, PROGRESS-Plus, Ireland

## Abstract

**Introduction:**

Organised screening reduces the incidence and late-stage diagnosis of cancer. However, participation in screening is not consistent across populations. Variations can be measured using demographic factors on place of residence, race/ethnicity, occupation, gender/sex, religion, education, socio-economic position (SEP), and social capital (PROGRESS-Plus stratifiers). The Republic of Ireland has screening programmes for colorectal, breast, and cervical cancer but assessment of screening participation and cancer incidence is inconsistent. The review aimed to evaluate the use of stratifiers in breast, cervical and colorectal cancer incidence and screening literature, and assess variations in incidence and screening participation across subgroups in Ireland.

**Methods:**

PubMed was searched systematically and grey literature was identified via Google, Google Scholar, Lenus (Irish Health Research repository), and The Irish Longitudinal Study of Aging (TILDA) in June 2022. Studies were included if they captured stratifiers alongside incidence or screening participation data of the three cancers.

**Results:**

Thirty-six studies and reports were included. Place of residence, SEP, sex, and age were most frequently captured. Incidence and screening participation varied by age, place of residence, SEP, and sex.

**Discussion:**

PROGRESS-Plus is a useful equity lens to review health literature. Cancer incidence and screening participation studies lacked a comprehensive equity lens resulting in difficulties in identifying inequities and non-attenders. Place of residence, SEP and ethnicity should be prioritised in monitoring inequities. Integrating unique health identifiers should improve monitoring and enable evidence-based population-specific interventions to promote screening. Collaboration with community organisations would support engagement with vulnerable populations when data is limited.

## Highlights

• Incidence of breast, cervical and colorectal cancer and participation in screening varies across subgroups in Ireland.
• Place of residence, socio-economic position, sex, and age were the most frequently captured stratifiers.
• PROGRESS-Plus is a useful equity lens to review health literature.
• Implementation of unique health identifiers would enhance equity monitoring and evidence-based promotion interventions.
• Collaboration with organisations who work with underscreened populations would support understanding of non-participation.

## 1 Introduction

### 1.1 Incidence of colorectal, breast and cervical cancer and participation in cancer screening

Europe accounted for almost a quarter (22.8%) of all cancers globally (World Health Organisation, 2020) and incidence is projected to rise over the coming decades (European Commission). In Europe, breast cancer is the most common cancer among women. Bowel cancer is the 3rd most common cancer and 2nd leading cause of cancer-related death for men and women in Europe. Cervical cancer is the 11th most common cancer for women. The incidence of these cancers varies across demographics such as age, sex, place of residence, socio-economic position and ethnicity (Buskwofie et al., 2020, Coughlin, 2019, Coughlin, 2020). The EU Beating Cancer Plan aims to reduce the burden of cancer and strive for equitable care across Europe (European Commission, 2022).

Cancer screening plays a key role in early identification of cancer and its pre-cursors, reducing morbidity and mortality (Raffle et al., 2019). Organised screening programmes invite certain groups in the population at intervals, based on evidence, to prevent or detect cancer early to reduce population morbidity cost-effectively (World Health Organisation, 2022). Alternatively, opportunistic screening is ad-hoc, relying on the individual to participate in screening. Differences in participation across subgroups in a population are lessened with organised compared to opportunistic screening (De Prez et al., 2022, De Prez et al., 2021). As with cancer incidence, participation in screening varies by demographic characteristics as well as contextual factors, such as family or provider recommendation and distance to the screening service (Rollet et al., 2021, Smith et al., 2019).

The EU recommends organised programmes for breast, colorectal and cervical cancer screening. The Republic of Ireland (Ireland) has three cancer screening programmes that are free to eligible populations: BowelScreen is available every two years to anyone aged between 60 and 69 (established in 2012); BreastCheck screens women aged 50–69 every two years (established in 2002); and CervicalCheck is available to people with a cervix aged 25–29 every 3 years and every 5 years to those aged 30–65 (established in 2008).

### 1.2 Measuring equity

Equity relates to fairness in health and can be defined as ‘*the absence of health disparities (and in its key social determinants) that are systematically associated with social advantage/disadvantage’* (Braveman and Gruskin, 2003) (p256). Inequity within this review refers to unfair disparities while inequality refers to differences in health across population groups (Arcaya et al., 2015). The social determinants of health consist of social, environmental, living and working conditions, social networks and individual lifestyle factors that influence access to healthcare and health outcomes (Dahlgren and Whitehead, 2021). Similarly, Cochrane PROGRESS-Plus is a list of factors that influence health called equity stratifiers. This includes: place of residence, race/ethnicity/culture/language, occupation, gender/sex, religion, education, socioeconomic position (SEP), social capital, and other characteristics including age, sexual orientation, disability, behaviours and relationships (Cochrane, Kavanagh et al., 2008). PROGRESS-Plus can be applied as an equity framework in health research (O'Neill et al., 2014).

There is an international push to understand and address equity in health (European European Commission, 2022, World Health Organisation, 2017). Monitoring health data can identify changes across the life course, enable comparisons across regions, identify disparities in health status, needs and access, and resource management (Tulchinsky and Varavikova, 2014).

### 1.3 Equity in the Irish context

Ireland has a mixed public and private health system. Some health services, such as GP consultations, incur fees and without a medical card (a means-tested entitlement to free public health services based on income, age, and health status) or private health insurance cover these are paid out-of-pocket. People who do not have proof of ordinary residence of at least 12 months, e.g., new migrants, are not eligible for medical cards or other entitlements regardless of income or health status. People of lower SEP and born outside of Ireland have lower life expectancy (Duffy et al., 2022). Over one-quarter of people in Ireland have limited health literacy and navigating the system can be challenging, especially for those with health conditions (National Adult Literacy Agency, 2020). The Health Service Executive (HSE) work with several vulnerable populations: people facing addiction, homelessness, domestic violence, intercultural communities, Irish Travellers and Roma populations, and lesbian, gay, bisexual, trans, queer, intersex people (LGBT+) (Health Service Executive, 2023). Recent health policies for vulnerable populations recognise the need for reform for appropriate care provision to address poorer health outcomes and inequitable access to services (Department of Health, 2022, Department of Housing Local Government and Heritage, 2016, National Social Inclusion Office, 2018, Policy Drugs Unit Social Inclusion, 2020, The Department of Justice and Equality. The Department of Justice and Equality, 2019). Irish Travellers, an ethnic minority population, exemplify a population with specific needs as they face discrimination in employment, housing and health service utilisation, as well as having poorer health outcomes (All Ireland Traveller Health Study Team, 2010, Department of Health, 2022). Additionally, the homeless population often have complex physical and mental care needs and struggle with addiction, have limited access to appropriate care, delay seeking care and rely on emergency services (Ní Cheallaigh et al., 2017). Sláintecare is a cross-party consensus health policy to reform the health system that strives for equitable, patient-centred, universal healthcare (Houses of the Oireachtas Committee on the Future of Healthcare, 2017). Slaintecare seeks to address the social determinants of health where patients are treated based on need rather than ability to pay and prevention and health promotion core tenets (Houses of the Oireachtas Committee on the Future of Healthcare, 2017).

Key to addressing the social determinants of health is measuring them. A recent study investigated what PROGRESS-Plus stratifiers were collected across health and social care data collections in Ireland (Carroll et al., 2021). Twenty-nine data dictionaries were included. Age, sex, place of residence and SEP were captured frequently, ethnicity, education, disability and sexual orientation less so. The Pobal HP Deprivation Index was the most commonly used measure for SEP which is a composite measure based on Central Statistics Office (CSO) area-level demographics that measures area-level population (not individual) affluence and deprivation (Haase and Pratschke, 2016). Overall, Ireland has limited means to capture equity, therefore synthesising literature is required to gain a clearer picture of health equity.

### 1.4 Aims

This study aimed to conduct a rapid review using a systematic approach to:

(1) Evaluate the use of equity measures in cancer incidence and cancer screening reports and quantitative studies in Ireland.
(2) Assess variations in incidence of colorectal, breast and cervical cancer across subgroups of the Irish population.
(3) Assess variations in participation in colorectal, breast, and cervical screening across subgroups of the Irish population.

## 2 Materials and methods

We conducted a systematic search of PubMed to identify peer-reviewed studies that address the aims of the review. We included grey literature such as needs assessments and reports from non-governmental organisations, the National Screening Service (NSS), National Cancer Registry Ireland (NCRI), and Central Statistics Office (CSO) regarding screening for colorectal, breast and cervical cancer.

We devised search strategies in PubMed for the three conditions on incidence and screening participation in Ireland, totalling six search strategies. The searches contained keywords including 'colorectal cancer’, ‘breast cancer’, ‘cervical cancer’, ‘incidence’, ‘screening’, ‘participat*’, ‘attend*’, ‘coverage’, ‘uptake’, ‘engage*’, ‘utilis*’, ‘non-attend*’, ‘non-respon*’, and ‘Ireland’, utilising the MeSH terms and title and abstract functions (Supplementary material 1). We did not limit the searches by time frame or language (Table 1). We ran the searches between May and June 2022. The most recently published NSS reports on screening participation, NCRI reports on cancer incidence and CSO health survey reports were deemed to have the most up-to-date information from these organisations and to provide the current baseline population-wide measures of screening participation and cancer incidence. We searched for additional literature and reports via Google, Google Scholar, and Lenus (Irish Health Research repository) searches, The Irish Longitudinal Study of Aging (TILDA) (a nationally representative longitudinal study) publication search tool and by reviewing the reference lists of the included sources.

**Table 1:** Inclusion and exclusion criteria.

**Inclusion**	**Exclusion**
Breast, cervical or colorectal cancer incidence	No data on cancer incidence
Breast, cervical or colorectal cancer screening participation	No data on screening participation
Data collected in the Republic of Ireland	Data collected outside of the Republic of Ireland
Collected and reported on demographic characteristics (i.e., PROGRESS-Plus stratifiers) PROGRESS Place of Residence (Rural/urban, county) Ethnicity (Ethnic background) Occupation (Professional, skilled, unskilled, unemployed etc.) Sex (Male or female) Religion (Religious background) Education (Years in and/or level of education attained) Social Capital (marriage, community/family support) Socio-economic position (SEP) (income, area-level deprivation or employment) PLUS Health cover/entitlements (Private health insurance, medical card) Age (Age range) Disability (physical or emotional/mental disability) Sexual orientation (Heterosexual, gay, lesbian, bisexual, transgender) Health status (poor/good health)	Did not collect or report on demographic characteristics
Reported in any language	
Published in any time frame	
Quantitative studies	Qualitative data

We included studies and reports if they; a) measured incidence of breast, cervical or colorectal cancer or b) measured breast, cervical or colorectal cancer screening participation; in the Republic of Ireland and; collected or reported PROGRESS-Plus stratifiers (Table 1). We excluded studies if they did not quantitively report on participation in breast, colorectal or cervical cancer screening; did not report incidence or screening participation in relation to any stratifier; were not in Ireland or segregated data on Ireland were not reported.

We downloaded search results into an Excel file. The lead authors screened titles and abstracts in Endnote 20 and tagged studies for inclusion or exclusion in an Excel file. SMS downloaded and screened full texts of the relevant studies. Uncertainties of inclusion or exclusion were resolved amongst the authors. We extracted data pertaining to peer-review status, study design, population, aims, methods, condition, use of stratifiers and results relevant to the research question into an Excel file (Supplementary material 2). PL extracted data for 20% of the included literature to check for concordance, of which there was high agreement. We synthesised studies narratively and categorised them by cancer type and incidence or screening participation, noting which PROGRESS-Plus equity stratifiers were used. A meta-analysis was not conducted due to the heterogeneity of data collection and analysis. SMS, appraised all peer-reviewed studies for quality using the Mixed Methods Appraisal tool (MMAT) (Hong et al., 2018), which appraises the appropriateness of methods for the research question, sample, measurements used, risk of bias and analytical methods of empirical studies for systematic reviews that include a variety of study types. For the quality appraisal of grey literature, Authority, Accuracy, Coverage, Objectivity, Date and Significance tool (AACODS) was used (Tyndall, 2010).

## 3 Results

### 3.1 Search results

**Fig. 1:**
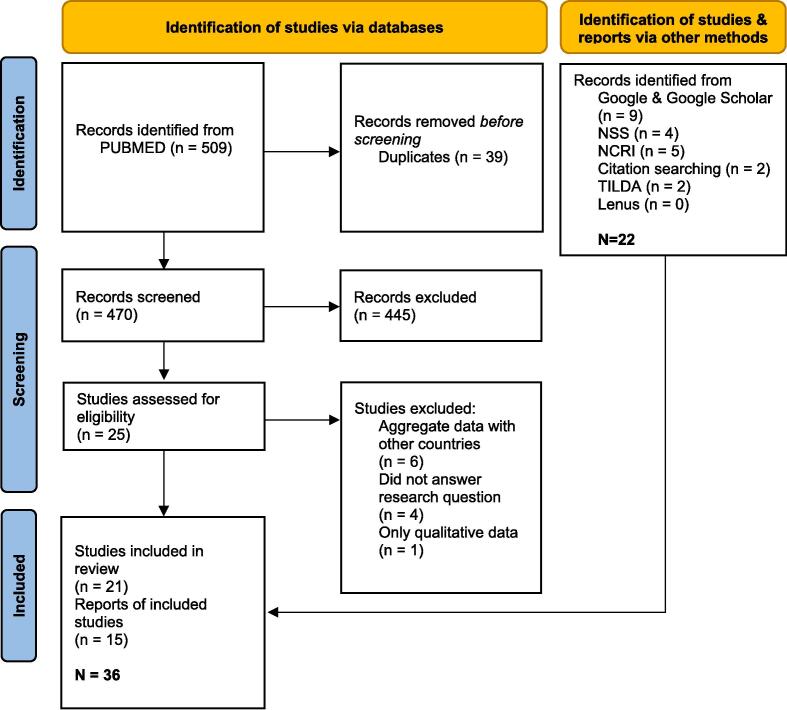
PRISMA flowchart of included studies and reports.

The PubMed searches yielded 509 results. After deduplication, 470 records remained, and 14 studies were included after full-text review. Twenty-two reports and studies from grey literature searching were identified and included. In total, Thirty-six studies and reports were included (Fig. 1). Twelve studies investigated incidence differences (Table 2) (Balhareth et al., 2018, Clarke et al., 2014, Donnelly et al., 2013, McDevitt et al., 2017, National Cancer Registry Ireland, 2018a, National Cancer Registry Ireland, 2022a, O'Brien and Sharp, 2013, Sharp et al., 2014, Ullah et al., 2018, Walsh et al., 2016). Twenty-four investigated differences in screening participation (All Ireland Traveller Health Study Team, 2010, Barrett et al., 2011, Central Statistics Office, 2020, Ch'ng et al., 2019, Clarke et al., 2016, Codd et al., 1994, Connolly and Whyte, 2019, Fitzpatrick et al., 2011, Fleming et al., 2013, Gallagher and Gallagher, 2010, Lalor and Redmond, 2009, McCarron et al., 2011, McNamara et al., 2014, McNamara et al., 2011, Moore et al., 2017, National Screening Service, 2020a, National Screening Service, 2020b, National Screening Service, 2021, National Screening Service, 2022, Ní Riain et al., 2001, O'Brien et al., 2018, Pavee Point Traveller and Roma Centre & Mid West Traveller Health Unit, 2019, Walsh et al., 2010, Walsh et al., 2012). Twenty studies reported on breast cancer, 16 on colorectal cancer and 15 on cervical cancer. Twenty-one were peer-reviewed studies, and 15 were reports published from the NSS, NCRI, CSO, TILDA, other reports included the All Ireland Traveller Health Study (AITHS) and one needs assessment on Traveller health. One study investigated incidence among a renal transplant patient population (Balhareth et al., 2018), all others used National Cancer Registry data. Studies on cancer screening measured participation by data from the national screening register or self-reported participation.

**Table 2:** Summary of included literature.

**Study**	**Population**	**Aims**	**Methods**	**Summary of findings**	**Quality Appraisal tool, score**
**Incidence**					
(National Cancer Registry Ireland, 2022a)	Registered colorectal, breast and cervical cancer population in Ireland.	Trends in **colorectal, breast and cervical** cancer incidence in Ireland 1994–2019.	National cancer registry age-standardised incidence rates (ASR incidence) calculated for breast, colorectal, cervical cancer using 2017–2019 data. Trends in age-standardised incidence rates 1994–2019 using joinpoint analysis. Age stratification based on the eligibility criteria of each screening programme.	Colorectal: ASR incidence 57.3 (male) and 37.7 (female) per 100,000. Overall decrease in incidence since 2012 (introduction of Colorectal Screening programme) for men and decrease for women since 1994. Decrease in incidence for men since 2012 for screening (60–69) and post-screening (70 + ) age groups, for women no change in trend.Breast: ASR incidence 130 (Female), 1.3 (male) per 100,000. Minor increase in incidence rates for women between 1994 and 2002 and 2014–2019. Women over 70 (post-screening age) incidence has increased since 1994.Cervical: ASR incidence 11.3 per 100,000. Significant decrease in incidence since 2009 (introduction of CervicalCheck). Incidence of cervical cancer increased then deceased between 1994 and 2018 for 25–60 (screening age) and has stayed the same for over 60′s (post screening age).	AACODS, high
(Walsh, McDevitt, Deady, O’Brien, & Comber, 2016)	Registered colorectal, breast and cervical cancer population in Ireland.	Inequalities in **colorectal, breast, cervical** cancers by age, sex, area deprivation and urban/rural status.	National cancer registry average estimates from 2008 to 2012. Age-standardised incidence rates (ASR incidence) calculated and association with demographic variables measured using rate ratios. Variables used: age, sex, area deprivation, urban–rural. Area-based measure of deprivation accounts for average socio-economic conditions within electoral divisions (EDs), based on 2006 census data (Pobal index*). Urban-rural status assigned based on theEDs where patients were resident at the time of their cancer diagnosis.	Colorectal: ASR incidence 17% higher for urban males than rural males, no difference in females. No significant relationship with area-deprivation or sex. Urban males showed significantly (13%) higher incidence between the most deprived and least deprived areas, not apparent in rural males, urban or rural females. Incidence increased with age, highest for over 55 years.Breast: ASR incidence were 13% higher in urban than in rural populations. Incidence rates were significantly lower (-15%) in the most deprived compared with the least deprived areas. For the oldest age group (75 + ), the incidence rate was 37% higher relative to age group 45–54. Urban populations had higher inequality in incidence by area deprivation compared to rural populations but was not statistically significant. Cervical: ASR incidence were 21% higher in urban compared to rural populations. Rates were 2.2 times higher in the most deprived compared with the least deprived areas, the most significant inequality by deprivation in the cancers analysed. Urban populations had highest differences in incidence by deprivation (+150% urban compared with 64% rural). Rates peaked 45–54 and 55–64 yrs.	AACODS, high
(Sharp et al., 2014)	Republic of Ireland (RoI) and Northern Ireland (NI) cancer population.	Urban rural variations in incidence of **18 cancers (incl. breast, colorectal and cervical**) in NI and RoI.	Relative risk of incidence calculated using binomial regression for 18 cancers. Cancers diagnosed between 1995 and 2007 extracted from the National Cancer Registry Ireland and Northern Ireland Cancer Registry. Investigated variation by urban rural living and adjusted for socioeconomic position (SEP). Areas based SEP calculated by area unemployment, area educational attainment, elderly living alone.	In RoI:Colorectal: Incidence higher in males. Relative risk significantly higher in both sexes in urban compared to rural areas (males 1.14, 1.09–1.18; females 1.04, 1.00–1.09) after SEP adjustment. Breast: Relative risk of breast cancer significantly higher in females in urban compared to rural areas after SEP adjustment. Cervical: Relative risk of cervical cancer significantly higher in females in urban compared to rural areas after SEP adjustment.	MMAT, high
(Donnelly et al., 2013)	Republic of Ireland (RoI) and Northern Ireland (NI) cancer population.	Comparison of incidence of **16 cancers (incl. breast, colorectal and cervical**) in NI and RoI and influence of age and socio-economic position.	229,824 cases for 16 cancers diagnosed in 1995–2007 were extracted from the National Cancer Registry Ireland and Northern Ireland Cancer Registry. Incidence rate ratios (IRR) calculated and compared by country and adjusted for age, sex, and area-based unemployment in electoral divisions (as proxy for area deprivation).	In RoI: Colorectal: Weak relationship between area deprivation and incidence for males only.Breast: Negative relationship between incidence and deprivation for breast (female) .Cervical: Cervical cancer rates lower in NI than in RoI after adjustment for age (IRR: 0.90 (0.84–0.97)), the difference increased after adjustment for SEP (IRR: 0.85 (0.79–0.92) ).	MMAT, high
(O'Brien & Sharp, 2013)	Irish cancer population.	Calculate **cervical** cancer incidence trends 1994–2008 and association with SEP.	National cancer registry data 1994–2008. Age-standardised incidence rate ratios calculated. Deprivation (SEP) based on characteristics from the 2002 census of the electoral district (ED) (Pobal index) the woman lived at the time of diagnosis and possession of a medical card +.	The mean age at diagnosis of women with cervical cancer was 48.6 years. Incidence increased over time 1994–2008. Incidence rate (invasive cancer) nearly double in most deprived compared to least deprived areas. Age-standardised rate was higher in those who held a medical card at the time of diagnosis compared to without a card, however it was not statistically significant.	MMAT, high
(National Cancer Registry Ireland, 2018b)	Irish cancer population.	Overview of **colorectal** cancer incidence, survival, and mortality	National cancer registry, Incidence calculated with 2015–2017 estimated averages.	Incidence rate 39.9 (females) and 65.2 (males) per 100,000 per year. Highest standardised incidence rate 1.06–1.15 in Cork, Louth, Longford, Leitrim. 36% cases in 75 + years and 31% in 65–74 years, median age group = 65–69.	AACODS, high
(National Cancer Registry Ireland, 2022b)	Irish cancer population.	Overview of **female breast** cancer incidence, survival, and mortality.	National cancer registry, Incidence calculated with 2017–2019 estimated averages.	Incidence rate 121.6 (invasive) 16 (in situ) per 100,000 per year. Highest standardised incidence rate 1.06–1.15 in Leitrim and Cork. 23% cases in ages < 50 and 41% cases in 50–64 years, median age group = 60–64.	AACODS, high
(National Cancer Registry Ireland, 2018a)	Irish cancer population.	Overview of **cervical** cancer incidence, survival, and mortality.	National cancer registry, Incidence calculated with 2015–2017 estimated averages.	Incidence rate 10.6 (invasive) 125.1 (in situ) per 100,000 per year. Highest standardised incidence rate 1.16 + in Wexford, Laois, Offaly, Westmeath. 58% cases in ages < 50, 25% cases in 50–64 years, Median age group = 45–49.	AACODS, high
(Clarke, McDevitt, Kearney, & Sharp, 2014)	Irish colorectal cancer population.	**Colorectal** cancer incidence 1994–2010.	National cancer registry Age-standardised incidence rates (ASR incidence) calculated from 1994 to 2010. Joinpoint regression for trend analysis.	ASR incidence 63.7 (males) and 38.7 (females) per 100,000 in 2010, little change in incidence over time. Over 65′s had highest incidence of cancer.	MMAT, high
(McDevitt, Comber, & Walsh, 2017)	Irish colorectal cancer population (n = 38,912).	Incidence of **colorectal** cancer 1994–2012.	National cancer registry, age-standardised incidence rate (ASR incidence) ratios of colorectal cancer between 1994 and 2012. Jotpoint for trend analysis.	ASR incidence was 65.2 (male) and 40.1 (females) per 100,000 between 2010 and 2012, did not changed over time.	MMAT, high
(Balhareth et al., 2018)	Renal transplant patients (n = 4731).	**Colorectal** cancer incidence in renal transplant patients between 1980 and 2007.	Retrospective review of a prospectively maintained database of all renal transplant recipients in Ireland between 1980 and 2017. Association between pathological outcomes and sex analysed using chi-square test.	Total of 33 patients developed colorectal cancer. More men than women developed colorectal cancer. The mean age at transplantation was 51.5 and 62.5 when diagnosed with colorectal cancer. Incidence rate was 62.35 per 100,000 in the renal transplant population. Infers based on international evidence that incidence (2–4 times) higher in transplant population than general population and tumours more advanced at diagnosis.	MMAT, high
(Ullah, Fleming, & Mealy, 2018)	Irish colorectal cancer population (n 39,528).	Incidence of **colorectal** cancer 1994–2012.	National cancer registry data on colorectal cancer 1994–2012, age-standardised incidence rates (ASR incidence) calculated and trends by regression analysis.	32.5% cases diagnosed between 70 and 79 years. Incidence increased 2.1% over time period. Incidence decreased for 50–79 but increased in 20–49 and 80 +.	MMAT, high
**Participation**					
(National Screening Service, 2020b)	Women eligible for breast screening (aged 50–69).	Assess coverage of **breast** screening in 2018–2019.	NSS summary data on screening participation in 2018 and 2019.	Coverage was 74.3% in 2018 and 71.6% in 2019 (70% target). Women invited for the first time attend at ∼ 70% but over 55 were less likely to attend. Subsequent participation is high at > 80% for all age groups. All counties achieved at least the 70% target coverage.	AACODS, high
(National Screening Service, 2022)	Women eligible for cervical screening (aged 25–60).	Assess coverage of **cervical** screening in 2017–2020.	NSS summary data on screening participation in 2017 and 2020.	Coverage 78.7% overall 2015–2020 5-year period (80% target). Less people participated in screening in 2018/19 year compared to 2017/18 – first time there’s been a decrease in coverage. Highest coverage in 25–29 age group. Coverage decreases with increasing age; 50–54 (71.8%), 55–59 (66.9%), 60+ (59.7%), a trend visible in previous years. Laois and Kilkenny had lowest coverage at < 70%. Colposcopy Did not attend rates under 10%.	AACODS, high
(National Screening Service, 2020a)	Men and women eligible for colorectal screening (aged 60–69).	Assess coverage of **colorectal** screening in 2018–2019.	NSS summary data on screening participation in 2018 and 2019.	Coverage at 41.9% (target 50%). Men have lower participation than women. Younger age (60–64) has higher participation at first invitation than older age (65–69). Subsequent participation is high at > 88%.	AACODS, high
(Clarke et al., 2016)	Individuals aged 50–74 living in an area in Dublin recruited from primary care centres (n = 9,785).	To assess participation in faecal immunochemical test (FIT) **colorectal** cancer screening.	Participants sent FIT test and information pack to participate in screening. Two rounds of screening 2008–2010 and 2011–2012. Poisson regression measured relative risk of participation of screening and association with sex, age, area deprivation (Pobal index).	Participation in both screening rounds combined was 60%; 41% of invitees took part in both rounds. Participation was lower in males compared to females. Younger age (under 60) and living in more deprived areas was associated with lower participation of screening.	MMAT, high
(McNamara, Qasim, Lee, Condon, & O’Morain, 2011)	Individuals aged 50–74 from primary care centres in an area of Dublin (n = 9,993).	To test the accuracy of the FIT test and evaluate the **colorectal** screening programme.	Pilot evaluation of the colorectal screening programme. Participation calculated by percentage (the number of returned FIT tests).	Participation was 51% in first round and 63% in second round. 58% (2,937) and 42% (2,126) of participants were women and men.	MMAT, moderate
(Connolly & Whyte, 2019)	Recruitment for the Irish Longitudinal study of aging (TILDA), women aged between 50 and 64 years (n = 6,902).	To examine the association between public healthcare eligibility and private health insurance (PHI) status on the participation of **breast** screening in over 50 year-olds.	TILDA survey, wave three data (2014–15). The association between eligibility and the participation of mammogram (breast screening) was analysed using multivariate logistic regression and odds ratios. Variables included: sex, age, marital status, employment, household income, location, health status, medical card, and PHI.	Overall participation for breast screening was 86%. Odds of participation higher for 55–59 and 60–64 year-olds, married women, higher education, higher income, higher for those with PHI only and PHI and medical cards, and those living in Dublin.	MMAT, high
(Central Statistics, 2020)	Sample of Irish population aged 15+ (n = 7,621).	To determine the health status of a sample of the Irish population including use of preventive health services (incl. **breast and cervical** screening).	CSO data self-reported health survey (cross-sectional) of representative sample of Irish population, invitation via household (cluster sampling and random sampling). Weighted results to represent the entire population and adjusted for non-response. Variables: region, age, employment, nationality, area deprivation (Pobal index).	Breast: 13% employed vs 6% unemployment had a mammogram in last 12 months. Lowest participation in Border and West regions (11%) and highest in South-East 15%. No major differences in other variables. Cervical: 27% non-Irish vs 19% Irish had cervical screening in last 12 months. Participation in last 12 months increased by increasing affluence (22% very affluent vs 17% very disadvantaged). Lowest participation was in the Border (16%) and South-East (15%) regions compared to Dublin (23%).	AACODS, high
(Ní Riain, Stewart, Phelan, Bury, & Mulcahy, 2001)	Five urban general practices (n = 395) and a genitourinary medicine (GUM) clinic (n = 323) (N = 718).	Difference between **cervical** screening knowledge, attitudes and practice in GP practices and a GUM clinic.	Cross-sectional self-report survey. 5 GP practices and a GUM clinic. Screening participation, purpose of screening and risk of cancer. Calculated percentages of survey responses. Variables: age, socio-economic position, employment nationality.	92% response rate at GP practices, 89% at GUM clinic. In the GP group, participation increased with increasing age and higher socio-economic position. 92% of GUM patients reported having a cervical screen in the last 3 years vs 75% in the GP sample.	MMAT, moderate
(Pavee Point Traveller, 2019)	Travellers in Co. Clare (n = 176).	Health needs assessment (inc. the use of preventive health services **Colorectal, Breast, Cervical** screening) of Travellers in Co Clare.	Survey and focus groups. 176 participants also gave information on 878 family members. Percentage participation of preventive health services in last 12 months.	Participants reported high participation of preventive health services. Colorectal: 4.3% participation Breast: 16% participation Cervical: 23% participation	AACODS, high
(National Screening Service, 2021)	Lesbian and bisexual women, trans men, non-binaryand intersex people with a cervix in Ireland (n = 418).	To explore knowledge attitudes, participation, and experiences of LGBT + people with **cervical** screening.	Cross-sectional self-reported online survey. Percentage participation of cervical screening. Variables: age, place of residence, education, ethnicity.	66.5% of participants said they attend every time they are invited. 26% are overdue (last screen over five years ago, never invited or invited but did not attend).	AACODS, high
(O'Brien, Mooney, Fitzpatrick, & Sharp, 2018)	Women aged 50–66 with breast cancer in Ireland (n = 7,161).	To investigate clinical and socio-demographic variables of **breast** cancer diagnosis that differ by differ by screening status between 2006 and 2011.	National Cancer Registry and national breast screening programme data were linked (2006–2011). Chi-squared and multinomial logistic regression were used to test the association of demographic variables with screening status. Variables: age, marital status, smoking status at diagnosis, area-deprivation (Pobal), co-morbidities; clinical variables were tumour subtype, stage, and grade of breast cancer.	Almost 40% of women did not participate in the screening programme, 43% had cancers which were screen-detected, 13% had interval cancers and 5% were lapsed attenders. The proportion of non-participants who were from the most deprived areas was the highest at 35%, compared to 30% for women with screen-detected cancer and lapsed attenders. Non-participants in screening were more likely to have at least one co-morbidity (OR = 1.50, 95% CI 1.17–1.92) and be from the most deprived area (OR = 1.21, 95% CI 1.02–1.42), age and smoking status were not associated with non-participants when compared to women with screen-detected cancer.	MMAT, high
(Barrett, Savva, Timonen, & Kenny, 2011)	Over 50 years old in Ireland. (n = 8,178), 52% women.	To determine the health and wellbeing (incl. **breast** screening participation) of people over 50 in Ireland.	The Irish Longitudinal Study on Ageing (TILDA). Nationally representative study of people aged 50 and over in Ireland. Stratified cluster sampling. Calculated percentage participation of breast screening. Variables: age, education, wealth, urban/rural.	73% women had a mammogram. Women in 50–54 age range more likely to have had a mammogram (related to introduction of national breast screening programme). Lower education and lower wealth associated with lower participation. Women in rural areas are more likely to self-examine and less likely to have a mammogram than urban women.	AACODS, high
(Fitzpatrick, Fleming, O'Neill, Kiernan, & Mooney, 2011)	Women aged 50–62 recalled for breast screening between 2000 nd 2007 (n = 11,765).	To quantify the impact of false-positive **breast** screening results on subsequent re-attendance.	Breast check data 2000–2007. Re-attendance compared between those with a false-positive recall to assessment and those not recalled to assessment. Chi-squared and *t* test and logistic regression analysis. Variables: age, assessment procedure, initial or subsequent screening, location of appointment for next screening round and time from recall to non-malignant diagnosis.	82.8% received a false-positive result. Women with false-positive results were more likely to attend for next screening compared to women not recalled. Invasive procedures, false-positive results at first screening, negatively impacted likelihood to attend next screening round. In logistic regression model first screening, older age, open surgical biopsy, appointment at a screening centre for next screening round and a longer wait for confirmation of non-malignant diagnosis were significant negative predictors of re-attendance at next screening round.	MMAT, high
(Walsh, Silles, & O'Neill, 2010)	Women aged 20–64 (cervical) RoI (n = 4402), NI (n = 1,764).;women aged 50–64 (breast) RoI (n = 1256), NI (n = 497).	To examine differences in the participation of **breast and cervical** screening related to socio-economic characteristics.	Cross-sectional surveys: SLAN 2007 survey in RoI and Northern Ireland Health and Social Wellbeing Survey 2005. Multivariate logistic regression. Screening participation in the last 12 months. Variables: socio-economic characteristics, occupation, and educational attainment.	Breast: Participation varied by education in RoI where women with low education had lower participation than women with higher education. Cervical: Participation varied by education in RoI same as for breast, participation was lower in the three lowest occupation groups compared to the highest levels of occupation group in RoI.	MMAT, high
(Walsh, Silles, & O'Neill, 2012)	Women aged 50–64 for breast (n = 1,203); women aged 25–64 for cervical (n = 3,937); individuals aged 50–74 (colorectal, pre-national programme) (n = 3,066).	To examine differences in the participation of **colorectal, breast, cervical** cancer screening in Ireland related to private medical insurance socio-economic position.	Cross-sectional survey, SLAN 2007 survey. Breast, colorectal and cervical screen in last 12 months. Decomposition analysis. Variables: Net equivalised household income, household socio-economic position (based on occupation), geographic location, age, marital status, education, possession of private insurance and self-reported health.	Colorectal: Higher income, higher socio-economic position, being female and having private insurance have greater participation of screening. 60% inequality between having and not having private insurance. Breast: Higher income, higher socio-economic position, higher educational attainment and having private insurance have greater participation of screening. 22% inequality between having and not having private insurance Cervical: Higher income, higher socio-economic position, higher educational attainment and having private insurance have greater participation of screening. Differences across socio-economic group is the largest determinant of inequality observed for cervical cancer.	MMAT, high
(Fleming, O'Neill, Owens, Mooney, & Fitzpatrick, 2013)	Women eligible for breast screening (50–69) (n = 1,797).	To determine the reasons why women skip rounds of **breast** screening and the factors influencing return of previous non-attenders (PNAs).	Chi-squared and logistic on breast and cervical attendance of regular attenders (controls) and first round and subsequent previous non-attenders (PNAs). Variables: age, socio-economic position, education, medical card holder, private insurance, urban/rural, family history of cancer, self-rated health.	Women living in rural areas, with below third level education, with private health insurance, and family with history of cancer were less likely to be in the first PNA group. Women living in rural areas were more likely to be in the subsequent PNA group to the control group.	MMAT, high
(All Ireland Traveller Health Study Team, 2010)	Travellers in Ireland (n = 143 breast; n = 141 cervical).	To capture the health (incl. participation in **breast and cervical** screening) of the Irish Traveller population.	Survey. Percentage participation of preventive services in the last 12 months.	Breast: in all ages, 25.2% reported attending for mammogram in the last 12 months, compared to 13.3% in the medical card population (SLAN 2007 data). Participation was higher in the 50–59 age group compared to the 60 + age group.Cervical: in all ages, 22.7% reported attending for cervical screening in the last 12 months compared to 11.6% from the medical card population in the SLAN study.	AACODS, high
(Ch'ng, Mooney, O'Donoghue, & Fitzpatrick, 2019)	Individuals aged 60–69 who received a negative (n = 105,276) or false-positive (n = 1,999) FIT (colorectal screen) in Ireland.	To quantify the impact of false-positive FIT in the first round of screening on re-attendance in **colorectal** screening.	Colorectal screening programme data in first round. Chi-squared, *t* test, Logistic regression. Variables: false-positive FIT, the effects of age group (≤64 or ≥ 65), gender, computed tomography colonography (CTC), biopsy, duration (in days) from bowel screening invitation to FIT result, and waiting time (in days) for colonoscopy appointment (after individuals were deemed suitable) on repeat participation in the second screening round.	Individuals with a negative FIT in the first round had a significantly higher participation in the second round (87.5%) than those who received a false-positive result in the first round (73.1%) and was similar across age groups and gender. Older age (>65), computed tomography colonography (unsuitability/failed colonoscopy) and longer duration from screening invitation to FIT result were predictors of non-re-attendance in the next screening round.	MMAT, high
(Moore, Scarlett, & Nolan, 2017)	Wave 3 of TILDA (n = 6,425).	To determine the healthcare utilisation (incl. **breast** screening) of people over 50 in Ireland.	The Irish Longitudinal Study on Ageing (TILDA). Nationally representative study of people aged 50 and over in Ireland. Stratified cluster sampling. Calculated percentage participation of breast screening. Variables: age, healthcare entitlement status (medical card cover) or private health insurance.	55% of older adult women in Ireland reported that they had a mammogram since their last interview. 88% of women aged 54–64 years (within the target population for breast screening) had a mammogram in the last two years. Healthcare entitlement status was associated with having a mammogram; those with ‘dual cover’ (29%) and a medical card or GP visit card only (42%) had the lowest rates, while those with PHI only (75%) and ‘no cover’ (77%) reported comparatively higher rates.	AACODS, high
(McNamara et al., 2014)	Individuals aged 50–75 from general practices in catchment area in Dublin (N = 9,863).	to assess key screening performance indicators during a second round of FIT-based **colorectal** screening in an Irish cohort and to compare results between successive rounds.	participation rate and odds ratios calculated as the percentage of individuals who were invited to take part in the programme and returned the FIT kits with either a negative or a positive result. Variables: age, sex.	Overall participation rate in round two of 47.5%. Women and older age associated with higher participation.	MMAT, low
(Codd et al., 1994)	Women aged 50–65 in several regions in Ireland (N = 29,115).	To evaluate the impact of a **breast** screening programme in Dublin.	Percentage participation. No statistical analysis plan detailed.	61% participation rate overall. Youngest age group (50–54) had highest participation.	MMAT, low
(Gallagher & Gallagher, 2010)	Women up to 60 years old in one general practice (N = 534).	Too audit one general practice's **cervical** screening rate before and after the introduction of a national cervical screening programme.	Cervical screening participation recorded at two time points and compared: October 1st 2008-July 31st 2009 and the same period in 2007/08. Variables: age.	Higher participation post-implementation of the national screening programme, 386 (72%) women participated in comparison to 148 (28%). Highest participation was in the 25–44 age group in both time periods and participation increase was greatest in this group.	MMAT, low
(Lalor & Redmond, 2009)	Primary carers of post-menopausal women with intellectual disabilities in three residential care settings (N = 129).	To determine practices of **breast** cancer screening among people with intellectual disabilities.	Survey. Percentage participation calculated. No statistical analysis plan detailed.	67% reported mammography screening. Lower percentage of received invitations and successful mammography for women with higher severity of intellectual disability.	MMAT, low
(McCarron, Burke, McGlinchey, & McCausland, 2011)	Random sample of 753 people aged over 40 with intellectual disability in Ireland, female (N = 415).	To understand the wellbeing of people with intellectual disabilities in Ireland, including healthcare utilisation (incl. **breast** screening) as they get older.	The Intellectual Disability Supplement to The Irish Longitudinal Study on Ageing. Random sampling. Descriptive analysis of variables of interest. Calculated percentage participation of self-reported screening.	40% of women over 40 years old attended breast screening. Screening participation decreased with greater severity of intellectual disability.	AACODS, high

*The Pobal index uses demographic profile, social class composition and labour market status from census data to create a standardised measure of deprivation in small areas in Ireland.

+ Medical card is a means-tested entitlement to free public health services based on income, age, and health status.

### 3.2 Critical appraisal

The majority of included studies were of high quality (Supplementary material 3). All grey literature was from reputable sources with clear aims, objectivity, and significance, however details of methodologies were often limited. Peer-reviewed studies were mostly of high quality. Some studies lacked transparency regarding the representativeness of samples, methodology or risk of non-response bias. No studies were excluded due to low quality.

### 3.3 Use of equity stratifiers

**Fig. 2:**
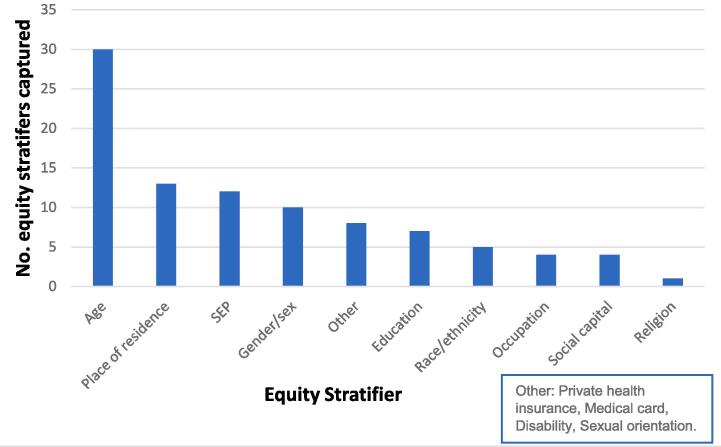
**Use of equity stratifiers in included literature**. SEP = Socio-economic position. Sex was only collected in colorectal cancer studies where screening is offered to males and females, gender identity was not captured.

The use of PROGRESS-Plus stratifiers varied widely. Sex, place of residence and SEP were the most commonly captured stratifiers, while religion was only captured in one study (Fig. 2; Tables 3a; Table 3b). Age was the most frequently captured stratifier. Some studies captured other stratifiers such as disability, sexual orientation, possession of private health insurance or a medical card. Incidence studies only used age, sex, place of residence and SEP stratifiers. Screening participation studies covered a broader range of stratifiers. Several studies measured SEP by the Pobal deprivation index, or otherwise occupation or area-based unemployment status (Donnelly et al., 2013, Sharp et al., 2014, Walsh et al., 2012). Despite capturing stratifiers, they were not always applied to determine variation across the population.

**Table 3a:** Table of use of equity stratifiers in screening participation studies by cancer type.

**Study**	**Place of residence**	**Ethnicity**	**Occupation**	**Sex**	**Religion**	**Education**	**Socio-economic position**	**Social capital**	**Age**	**Other**
**Breast**										
(National Cancer Registry Ireland, 2022a)									✓	
(Walsh, McDevitt, Deady, O’Brien, & Comber, 2016)	✓						✓		✓	
(Sharp et al., 2014)	✓						✓			
(Donnelly et al., 2013)							✓		✓	
(National Cancer Registry Ireland, 2022b)	✓								✓	
**Cervical**										
(National Cancer Registry Ireland, 2022a)									✓	
(Walsh et al., 2016)	✓						✓		✓	
(Sharp et al., 2014)	✓						✓			
(Donnelly et al., 2013)							✓		✓	
(O'Brien & Sharp, 2013)							✓		✓	✓^2^
(National Cancer Registry Ireland, 2018a)	✓								✓	
**Colorectal**										
(National Cancer Registry Ireland, 2022a)				✓					✓	
(Sharp et al., 2014)	✓			✓			✓			
(Walsh et al., 2016)	✓			✓			✓		✓	
(Donnelly et al., 2013)				✓			✓		✓	
(National Cancer Registry Ireland, 2018b)	✓			✓					✓	
(Clarke, McDevitt, Kearney, & Sharp, 2014)				✓					✓	
(McDevitt, Comber, & Walsh, 2017)				✓					✓	
(Balhareth et al., 2018)				✓					✓	
(Ullah, Fleming, & Mealy, 2018)									✓	
**Total**	**9**	**0**	**0**	**8**	**0**	**0**	**10**	**0**	**17**	**0**

1. Private health insurance; 2. Medical card; 3. Disability; 4. Sexual orientation.

**Table 3b:** Table of use of equity stratifiers in screening participation studies by cancer type.

**Study**	**Place of residence**	**Ethnicity**	**Occupation**	**Sex**	**Religion**	**Education**	**Socio-economic position**	**Social capital**	**Age**	**Other**
**Breast**										
(National Screening Service, 2020b)	✓								✓	
(Connolly & Whyte, 2019)	✓		✓			✓	✓	✓	✓	✓^1,2^
(Central Statistics, 2020)	✓	✓	✓				✓		✓	
(Pavee Point Traveller and Roma Centre & Mid West Traveller Health Unit, 2019)		✓								
(O'Brien, Mooney, Fitzpatrick, & Sharp, 2018)							✓	✓	✓	
(Barrett, Savva, Timonen, & Kenny, 2011)	✓					✓	✓		✓	
(Fitzpatrick, Fleming, O'Neill, Kiernan, & Mooney, 2011)									✓	
(Walsh, Silles, & O'Neill, 2010)			✓			✓				
(Walsh, Silles, & O'Neill, 2012)						✓	✓	✓	✓	✓^1^
(Fleming, O'Neill, Owens, Mooney, & Fitzpatrick, 2013)	✓					✓	✓			✓^2^
(All Ireland Traveller Health Study Team, 2010)		✓							✓	
(Moore, Scarlett, & Nolan, 2017)									✓	✓^1,2^
(Codd et al., 1994)									✓	
(Lalor & Redmond, 2009)										✓^3^
(McCarron, Burke, McGlinchey, & McCausland, 2011)	✓			✓	✓	✓		✓	✓	✓^3^
**Cervical**										
(National Screening Service, 2022)	✓								✓	
(Central Statistics, 2020)	✓	✓	✓				✓		✓	
(Ní Riain, Stewart, Phelan, Bury, & Mulcahy, 2001)		✓	✓				✓		✓	
(Pavee Point Traveller and Roma Centre & Mid West Traveller Health Unit, 2019)		✓								
(National Screening Service, 2021)	✓	✓				✓			✓	✓^4^
(Walsh, Silles, & O'Neill, 2010)			✓			✓				
(Walsh, Silles, & O'Neill, 2012)						✓	✓	✓	✓	✓^1^
(All Ireland Traveller Health Study Team, 2010)		✓							✓	
(Gallagher & Gallagher, 2010)									✓	
**Colorectal**										
(National Screening Service, 2020a)				✓					✓	
(Clarke et al., 2016)				✓			✓		✓	
(McNamara, Qasim, Lee, Condon, & O’Morain, 2011)				✓						
(Pavee Point Traveller and Roma Centre & Mid West Traveller Health Unit, 2019)		✓								
(Walsh, Silles, & O'Neill, 2012)				✓		✓	✓	✓	✓	✓^2^
(Ch'ng, Mooney, O'Donoghue, & Fitzpatrick, 2019)				✓					✓	
(McNamara et al., 2014)				✓					✓	
**Total**	**9**	**9**	**6**	**7**	**1**	**10**	**11**	**6**	**23**	**9**

1. Private health insurance; 2. Medical card; 3. Disability; 4. Sexual orientation.

### 3.4 Incidence results

Variations in incidence by PROGRESS-Plus stratifier are summarised in Table 4.

**Table 4:** Variations in incidence and screening participation by PROGRESS-Plus Stratifiers.

**Cancer**	**Place of residence**	**Ethnicity**	**Occupation**	**Sex**	**Religion**	**Education**	**Socio-economic position**	**Social capital**	**Age**	**Other**
**Incidence**										
**Breast**	↑ urban ↕ county	ND	ND	ND	ND	ND	↓ low SEP	ND	↑ 60–64	ND
**Cervical**	↑ urban ↕ county	ND	ND	NA	ND	ND	↑ low SEP	ND	↑ 45–49	ND
**Colorectal**	↑ urban ↕ county	ND	ND	↑ male	ND	ND	↕ low SEP	ND	↑ 64–69	ND
**Screening Participation**										
**Breast**	↕ urban ↕ county	- Irish ↓ Traveller	↓ unemployed	NA	ND	↓ low Ed	↓ low SEP	↑ married	↑ 55+	↑ PHI ↕ Medical card ↓ Disability
**Cervical**	ND urban ↕ county	↓ Irish ↓ Traveller	ND	NA	ND	↓ low Ed	↓ low SEP	ND	↓ 50+	↓ LGBT+ ↑ PHI
**Colorectal**	ND	↓ Traveller	- unemployed	↓ male	ND	ND	↓ low SEP	ND	↕	ND

ND = No Data found; ↕ = results varied/inconclusive; ↓ = decreased; ↑ = increased; - = no difference; NA = Not applicable. SEP = Socio-economic position, Ed = education, PHI = Private health insurance, LGBT+ = Sexual orientation.

#### 3.4.1 Colorectal cancer

##### 3.4.1.1 Sex

The average age-standardised incidence rates of colorectal cancer were 57.3 (male) and 37.7 (female) per 100,000 between 2017 and 2019 in Ireland (National Cancer Registry Ireland, 2022a). Males consistently had higher incidence of colorectal cancer (Balhareth et al., 2018, Clarke et al., 2014, McDevitt et al., 2017, National Cancer Registry Ireland, 2018b, National Cancer Registry Ireland, 2022a, Sharp et al., 2014, Walsh et al., 2016).

##### 3.4.1.2 Age

Increased incidence of colorectal cancer was significantly associated with increasing age (Clarke et al., 2014, National Cancer Registry Ireland, 2018b, National Cancer Registry Ireland, 2022a, Ullah et al., 2018, Walsh et al., 2016). According to NCRI, the 75+ age group account for 36% of cases and 31% in 65–74 age group, with 65–69 being the median age group (National Cancer Registry Ireland, 2018b).

##### 3.4.1.3 Place of residence

The highest standardised incidence rate of 1.06–1.15 were in counties Cork, Louth, Longford, and Leitrim. (National Cancer Registry Ireland, 2018b). Increased incidence was associated with living in urban areas, however in one study this association was only evident in males (Sharp et al., 2014, Walsh et al., 2016).

##### 3.4.1.4 Socio-economic position

Area deprivation was used to measure SEP. One study found a weak association with more deprived areas and higher incidence of colorectal cancer in males only (Donnelly et al., 2013). In another study, an association was only seen among urban males who showed significantly higher incidence in more deprived areas (13%), this association was not evident among females or in rural areas (Walsh et al., 2016).

#### 3.4.2 Breast cancer

The average age-standardised incidence rate for female only breast cancer was 121.6 per 100,000 (National Cancer Registry Ireland, 2022a). All studies reported data for female breast cancer.

##### 3.4.2.1 Age

Incidence of breast cancer increased significantly with increasing age, the median age group according to NCRI was 60–64 years (National Cancer Registry Ireland, 2022b). The association between older age and higher incidence of breast cancer was evident in all three studies that reported on it (National Cancer Registry Ireland, 2022a, Walsh et al., 2016).

##### 3.4.2.2 Place of residence

The counties with the highest standardised incidence rate (1.06–1.15) were Leitrim and Cork (National Cancer Registry Ireland, 2022b). Incidence was higher in urban areas (Sharp et al., 2014, Walsh et al., 2016).

##### 3.4.2.3 Socio-economic position

Women from the least deprived areas were more likely to be diagnosed with breast cancer (Donnelly et al., 2013, Walsh et al., 2016). One study determined that differences in breast cancer incidence between most deprived and least deprived areas were greater in urban compared to rural areas (Walsh et al., 2016).

#### 3.4.3 Cervical cancer

The average age-standardised incidence rate for cervical cancer was 11.3 per 100,000 between 2017 and 2019 (National Cancer Registry Ireland, 2022a).

##### 3.4.3.1 Age

Women between 25 and 59 had higher incidence at 19.1 per 100,000 compared to older age groups (National Cancer Registry Ireland, 2022a) with the majority of cases occurring under 50 years of age and the median age group was 45–49 (National Cancer Registry Ireland, 2018a, O'Brien and Sharp, 2013, Walsh et al., 2016).

##### 3.4.3.2 Place of residence

The counties with the highest standardised incidence rate of 1.16+ per 100,000 were Westmeath, Laois, Offaly and Wexford (National Cancer Registry Ireland, 2018a). Incidence was higher in urban areas (Sharp et al., 2014, Walsh et al., 2016).

##### 3.4.3.3 Socio-economic position

Cervical cancer incidence had the strongest association with area deprivation compared to the other cancers, with incidence nearly double in the most compared to least deprived areas (O'Brien and Sharp, 2013, Walsh et al., 2016). One study found differences between most deprived and least deprived areas were greater in urban compared to rural areas (Walsh et al., 2016). One study used the medical card as a variable to explore incidence on cervical cancer and found that medical card holders had higher incidence of the disease, although it was not statistically significant (O'Brien and Sharp, 2013).

#### 3.4.4 Use of other stratifiers in incidence differences

No studies or reports analysed the association of ethnicity, religion, education, occupation or social capital on breast, cervical and colorectal cancer incidence. Equally, none captured data on the Traveller population, people with disabilities, homeless people, or the LGBT+ community.

### 3.5 Participation results

Variations in participation are summarised in Table 4.

#### 3.5.1 Colorectal screening

Colorectal screening participation of the eligible population was 41.9% in the 2018–2019 interval, and has steadily increased since it was introduced in 2012 (National Screening Service, 2020a).

##### 3.5.1.1 Sex

Men were less likely than women to participate in colorectal screening (Clarke et al., 2016, McNamara et al., 2014, McNamara et al., 2011).

##### 3.5.1.2 Age

Men and women in the older age bracket (65–69) eligible for screening had lower participation rates for colorectal screening in one report (National Screening Service, 2020a). However, two studies found older age was associated with higher participation (Clarke et al., 2016, McNamara et al., 2014). Older age also contributed to reduced likelihood of return to colorectal screening after false-positive results compared to individuals with negative results (Ch'ng et al., 2019).

##### 3.5.1.3 Place of residence

No data were available on county-level differences (National Screening Service, 2020a) or urban–rural place of residence.

##### 3.5.1.4 Socio-economic position

There was an association between deprivation and participation, whereby participation was higher in least deprived areas in one area in Dublin (Clarke et al., 2016). Higher income, higher SEP, and having private insurance were associated with greater participation before the national programme was implemented (Walsh et al., 2012).

##### 3.5.1.5 Ethnicity

A study conducted among the Traveller community living in County Clare reported 4.3% participation of colorectal cancer screening in the previous 12 months (Pavee Point Traveller and Roma Centre & Mid West Traveller Health Unit, 2019). No other studies reported on ethnicity.

##### 3.5.1.6 Use of other stratifiers

No studies reported on differences in participation of colorectal screening by occupation, education, religion, or social capital.

#### 3.5.2 Breast screening

Breast screening achieved 71.6% participation in a two-year cycle (2018–19) (National Screening Service, 2020b).

##### 3.5.2.1 Age

Women in the older eligible age bracket (55–64) had high participation rates for breast screening (Connolly and Whyte, 2019), but participation at first invitation declined with increasing age (National Screening Service, 2020b).

##### 3.5.2.2 Place of residence

All counties reached at least 70% participation over the 2018–19 interval for breast screening (National Screening Service, 2020b). A 2019 CSO survey reported that self-reported participation in the last 12 months, adjusted to represent the population over 15 years old, was lowest in Border and West regions (11%) and highest in South-East (15%) (Central Statistics Office, 2020). In another study, living in Dublin was associated with increased likelihood of participating compared to rural areas or other towns and cities (Connolly and Whyte, 2019). Similar urban–rural differences were evident in a study on previous non-attenders of breast screening (Fleming et al., 2013). In contrast, a study from 2011 using the first wave of TILDA data, reported women (aged 50+) in rural areas were less likely to have a mammogram than urban women (Barrett et al., 2011).

##### 3.5.2.3 Socio-economic position, education, occupation

The CSO reported mammogram self-reported participation in the last 12 months at 13% for employed women compared to 6% among unemployed women but no difference by area deprivation (Central Statistics Office, 2020). The odds of participation in breast screening increased with higher educational attainment, higher income, and higher SEP (Barrett et al., 2011, Connolly and Whyte, 2019, Walsh et al., 2010, Walsh et al., 2012).

##### 3.5.2.4 Ethnicity

A minor difference was evident between Irish (13%) and non-Irish (10%) nationality for self-reported participation at breast screening in the CSO survey (Central Statistics Office, 2020).

In a 2019 needs assessment of Travellers living in Clare, 16% self-reported breast screening participation in the last 12 months; this was lower than the comparable 2010 AITHS study of 25% (All Ireland Traveller Health Study Team, 2010, Pavee Point Traveller and Roma Centre & Mid West Traveller Health Unit, 2019).

##### 3.5.2.5 Use of other stratifiers

Participation increased for women with private medical insurance (Connolly and Whyte, 2019, Moore et al., 2017, Walsh et al., 2012). Possession of a medical card reduced participation in screening but possession of both a medical card and private health insurance was associated with higher participation in another study (Connolly and Whyte, 2019, Moore et al., 2017).

The odds of participation in breast screening were higher for married women and those in good health (Connolly and Whyte, 2019).

Two studies that investigated intellectual disabilities in Ireland found that participation was lower than the general population and participation decreased with more severe disability (Lalor and Redmond, 2009, McCarron et al., 2011).

Social capital was captured only by marriage. Religion was the only equity stratifier not captured.

#### 3.5.3 Cervical screening

Cervical screening participation of the eligible population was 78.7% during the 2015–2020 screening interval (National Screening Service, 2022).

##### 3.5.3.1 Age

Of the eligible population, younger women had the highest participation in the 5-year period 2015–2020, which declined with increasing age (50–54, 71%; 55–59, 67%; 60+, 59%), a trend visible since the introduction of the cervical screening programme in 2008 (National Screening Service, 2022). A study in one general practice corroborated this finding with women aged 25–44 having highest participation in screening (Gallagher and Gallagher, 2010).

##### 3.5.3.2 Place of residence

Counties Laois and Kilkenny had the lowest participation rate for cervical screening at 69%, while Clare, Dublin, Monaghan and Roscommon all had participation below 75% in the 2015–2020 interval (National Screening Service, 2022). No data were found on urban–rural place of residence and cervical screening participation. The 2019 CSO Health Survey found lowest self-reported cervical screening participation in the last 12 months, among the population over 15 years old, in the South-East (15%) region for cervical screening compared to the highest rates in Dublin (23%) (Central Statistics Office, 2020).

##### 3.5.3.3 Socio-economic position, education, occupation

Most studies that analysed socio-economic variation pre-date the establishment of CervicalCheck. Participation varied by education, whereby women with lower levels of educational attainment had lower participation (Walsh et al., 2010, Walsh et al., 2012). In one study where occupation was used to measure SEP, lower SEP had lower odds of participation (Walsh et al., 2010). Higher income, higher SEP, and having private insurance were associated with greater participation in screening (Walsh et al., 2012). In another study, participation increased with higher SEP in the GP patient population (Ní Riain et al., 2001). Participation in the last 12 months was lower in the most deprived compared to least deprived areas among the population over 15 years old (Central Statistics Office, 2020).

##### 3.5.3.4 Ethnicity

Twenty-seven percent of non-Irish women reported having cervical screening in the last 12 months compared to 19% of Irish women in the CSO report that surveyed people aged 15+ (beyond the eligible screening age) (Central Statistics Office, 2020).

In another 2019 study, self-reported cervical screening participation in the last 12 months among Travellers in Clare was high and remained the same as the 2010 AITHS at 23% (All Ireland Traveller Health Study Team, 2010, Pavee Point Traveller and Roma Centre & Mid West Traveller Health Unit, 2019).

##### 3.5.3.5 Use of other stratifiers

In a study conducted with the LGBT+ community, 66% of respondents said they attended every time they were invited (i.e., every 3–5 years), lower than the national rate of 78%. 20% reported that their last test was over 5 years ago or they had never been screened (National Screening Service, 2021).

No studies analysed participation of cervical screening by religion or social capital. No studies investigated non-attender demographics for cervical cancer screening.

## 4 Discussion

This review evaluated the use of PROGRESS-Plus stratifiers in breast, cervical and colorectal cancer incidence and screening participation literature and mapped differences in incidence and screening participation across subgroups in Ireland. PROGRESS-Plus was a useful framework to monitor health equity. The findings highlight a limited use of stratifiers and variations in methodologies, resulting in difficulties determining true subgroup differences in screening participation.

### 4.1 Measuring equity in cancer incidence

Incidence studies captured a limited set of stratifiers to understand subgroup differences in colorectal, breast and cervical cancer. Sex, age, place of residence and SEP were the only demographics used to explore inequities and was limited to data from the National Cancer Registry. The results from this review are comparable to trends in variation by SEP (Hiscock et al., 2012, Mihor et al., 2020) and urban areas (Carnegie et al., 2022). Despite male breast cancer being rare, it is important to capture sex to ensure the needs of this underserved population can be met (Fentiman, 2018). Late stage diagnosis and increased mortality is evident for those in more deprived areas in colorectal, breast and cervical cancer adding to disparities (Bambury et al., 2023, Walsh et al., 2016). Consistent measurement of sex, SEP and place of residence in relation to incidence and other outcomes would support resource allocation required for equitable access to care.

### 4.2 Measuring equity in cancer screening participation

Studies and reports varied widely in capturing screening participation. Some studies used convenience sampling, others had nationally representative samples or used the screening registry data, this limited the ability to compare results as some were prone to selection bias. Future research would benefit from standardising reporting participation in line with the screening intervals, ideally linking to screening registry data rather than using self-report data as recall bias influences the accuracy of the results.

Variations across stratifiers was more broadly captured in participation literature but poorly captured for non-attender demographics. Breast screening was most commonly studied and comprised the widest use of equity stratifiers. Screening registry data is limited to age, sex, and county level stratifiers and would benefit from including SEP and ethnicity stratifiers. The trends in screening participation align with international studies where education, age, income, place of residence, and disability are all determinants (Carrozzi et al., 2015, Dugord and Franc, 2022, McCowan et al., 2019, Mihor et al., 2020, Walji et al., 2021). The lower participation of men in colorectal screening is evident internationally (Clarke et al., 2015). We postulate whether women’s engagement with breast and cervical screening raises their engagement with colorectal screening compared to men. High participation, particularly cervical screening, among Travellers was attributed to primary healthcare projects and community health workers (Department of Health, 2022). LGBT+ participation was lower than the national average, likely an underestimate given the sample had few older women and people with lower educational attainment; the main barriers to participating include heteronormative assumptions being made by healthcare professionals and fear of the test procedure (National Screening Service, 2021). In one study, non-Irish women were identified as having higher levels of participation in cervical screening, contrary to most research that indicates participation varies due to cultural differences in attitudes and access to care by ethnicity and migration status (Anderson de Cuevas et al., 2018, Marlow et al., 2015, Tatari et al., 2020). It is not clear what the reason for this result is, however cultural norms where women may get screened annually go for private screens with gynaecologists or attend in their home countries could be a factor (Marques et al., 2020, Marta et al., 2012). Further work should identify non-attenders, their barriers and enablers to participation, and intersectional interactions between stratifiers.

### 4.3 Enhancing monitoring of cancer incidence and screening participation data

Digital health monitoring is needed to reduce inequities in mortality and morbidity of cancer. As part of the EU Beating Cancer Plan, a cancer inequalities registry has been created to track variations across countries but is only as good as the data they receive (European Commission, 2022). The HSE are striving to enhance ethnicity monitoring across health services and the NSS are planning to introduce geo-coded data to the screening registry. Individual health identifiers, initially recommended over ten years ago, are vital to integration of health information systems and plans are underway to implement them in screening registries (Health Information and Quality Authority, 2011). These are valuable but small steps towards improved monitoring. Ireland has strategies to improve information systems, however implementation has been difficult due to resourcing issues and political pressure is required to enable whole system changes (Health Service Executive, 2013, Health Service Executive, 2015, Walsh et al., 2021). Countries such as Scotland and Denmark have systems to monitor health, collect equity stratifers and are used in planning and research (Walsh et al., 2021). The World Health Organisation provide a 5-step manual for best practice in managing health inequalities: 1) determining the scope of monitoring, 2) obtain data, 3) analyse data, 4) report results, 5) implement changes (World Health Organisation, 2017). The manual provides a framework to support Irish and other health systems experiencing shortcomings in digital transformation and facilitate cross-country comparisons.

Equity-focused research will provide evidence to address inequities in health outcomes, service provision and access. PROGRESS-Plus captured the social determinants of health well and has scope to capture determinants specific to Ireland including medical cards, private health insurance, and determinants relevant to vulnerable populations. Understanding the needs of vulnerable groups who have more issues accessing services can determine where inequities exist. Indeed, some literature included in this review conducted research with vulnerable groups (All Ireland Traveller Health Study Team, 2010, National Screening Service, 2021, Pavee Point Traveller and Roma Centre & Mid West Traveller Health Unit, 2019). This work could be replicated with other groups who have typically lower participation in screening and engagement with other health services. Qualitative research would be valuable in determining factors influencing non-participation, beliefs and attitudes towards cancer and screening. Other underlying social determinants of health not captured in PROGRESS-Plus, such as cultural influence, knowledge, or practical constraints (e.g. neighbourhood facilities, access to services), contributing to participation would be illuminated in qualitative research and theory (Chorley et al., 2017, Le Bonniec et al., 2023). The qualitative study excluded at full text in this review found differing influences of participation in older and younger women using behaviour theory framework to contribute to developing population-specific interventions (O'Donovan et al., 2021). There is a plethora of intervention studies that effectively promote screening among underscreened populations that provide insights on engagement with these populations (Cullerton et al., 2016, Dietrich et al., 2006, Dunn et al., 2017). Efforts should focus on research with stakeholder engagement and collaboration with community organisations to develop evidence-based interventions for underscreened populations.

### 4.4 Limitations

This review was conducted rapidly as part of research to inform the development of a strategic framework to improve equity in screening for the National Screening Service (unpublished) due to time and resource constraints. Hence, only one database was searched and there was limited consultation with stakeholders to identify other relevant literature. Screening and cross-checking data extraction by multiple authors was completed for a sample of all included literature, potentially missing evidence valuable to the review. Meta-analysis was not possible because the included data was not compatible with this approach. Some included studies predated the introduction of the screening programmes, therefore stratifiers influencing participation may have changed since then. This review excluded qualitative studies, exploration of the barriers and facilitators influencing participation in screening was not possible.

## 5 Conclusion

Gaps in the measurement of equity for breast, colorectal and cervical cancer incidence and participation in screening were identified. PROGRESS-Plus is a useful equity lens to review health literature. Particular attention should be paid to consistently monitoring place of residence, ethnicity, and SEP while efforts should be made to capture lesser used stratifiers. Implementation of unique health identifiers and integration of health datasets is required to progress equity of access to care and cancer outcomes. Moving away from self-reported data on screening and linking screening registry data to uncover variations across stratifers would enhance evidence to prioritise resources and implement tailored interventions that promote screening where it is needed. Qualitative research would shed light on underlying influences of participation, beliefs and attitudes towards cancer and screening. Leveraging relationships with community organisations may support understanding of challenges within specific non-attender populations who are hard to engage with.

## Declaration of Competing Interest

The authors declare that they have no known competing financial interests or personal relationships that could have appeared to influence the work reported in this paper.

## Data Availability

No data was used for the research described in the article.

## References

[b0005] All Ireland Traveller Health Study Team. 2010. All Ireland Traveller health study: Summary of findings. Available from: https://www.gov.ie/en/publication/b9c48a-all-ireland-traveller-health-study/.

[b0010] Anderson de Cuevas, R.M., Saini, P., Roberts, D., Beaver, K., Chandrashekar, M., Jain, A., Kotas, E., Tahir, N., Ahmed, S., et al., 2018. A systematic review of barriers and enablers to South Asian women’s attendance for asymptomatic screening of breast and cervical cancers in emigrant countries. BMJ Open 8:e020892. DOI:10.1136/bmjopen-2017-020892. 10.1136/bmjopen-2017-020892 PMC6042536 29982210

[b0015] Arcaya M.C., Arcaya A.L., Subramanian S.V. (2015). Inequalities in health: definitions, concepts, and theories. Glob. Health Action.

[b0020] Balhareth A., Reynolds I.S., Solon J.G., Harte E.G., Boland F., O'Sullivan J.M., Burke J.P., Little D., McNamara D.A. (2018). Thirty-seven-year population-based study of colorectal cancer rates in renal transplant recipients in Ireland. Transpl. Proc..

[b0025] Bambury N., Brennan A., McDevitt J., Walsh P. (2023).

[b0030] Barrett, A., Savva, G., Timonen, V., Kenny, R. 2011. Fifty Plus in Ireland 2011. First results from the Irish Longitudinal Study on Ageing (TILDA). Trinity College Dublin, Ireland Report No.: ISBN: 978-1-907894-01-5 Available from: https://tilda.tcd.ie/publications/reports/pdf/w1-key-findings-report/Tilda_Master_First_Findings_Report.pdf.

[b0035] Braveman P., Gruskin S. (2003). Defining equity in health. J. Epidemiol. Community Health.

[b0040] Buskwofie A., David-West G., Clare C.A. (2020). A review of cervical cancer: incidence and disparities. J. Natl Med. Assoc..

[b0045] Carnegie E.R., Inglis G., Taylor A., Bak-Klimek A., Okoye O. (2022). Is population density associated with non-communicable disease in western developed countries? a systematic review. Int. J. Environ. Res. Public Health.

[b0050] Carroll C., Evans K., Elmusharaf K., O’Donnell P., Dee A., O’Donovan D., Casey M. (2021). A review of the inclusion of equity stratifiers for the measurement of health inequalities within health and social care data collections in Ireland. BMC Public Health.

[b0055] Carrozzi G., Sampaolo L., Bolognesi L., Sardonini L., Bertozzi N., Giorgi Rossi P., Zappa M., Baldissera S., Campostrini S. (2015). Cancer screening uptake: association with individual characteristics, geographic distribution, and time trends in Italy. Epidemiol. Prev..

[b0060] Central Statistics Office. 2020. Health Determinants [Available from: https://www.cso.ie/en/releasesandpublications/ep/p-ihsmr/irishhealthsurvey2019-mainresults/healthdeterminants/.

[b0065] Ch'ng B.X., Mooney T., O'Donoghue D., Fitzpatrick P. (2019). Return to bowel screening after a false-positive faecal immunochemical test in BowelScreen (the National Bowel Screening Programme in Ireland). J. Med. Screen..

[b0070] Chorley A.J., Marlow L.A., Forster A.S., Haddrell J.B., Waller J. (2017). Experiences of cervical screening and barriers to participation in the context of an organised programme: a systematic review and thematic synthesis. Psychooncology.

[b0075] Clarke, N., Sharp, L., Osborne, A., Kearney, P.M., 2015. Comparison of uptake of colorectal cancer screening based on fecal immunochemical testing (FIT) in males and females: a systematic review and meta-analysis. Cancer Epidemiol Biomarkers Prev 24:39-47. DOI:10.1158/1055-9965.Epi-14-0774. 10.1158/1055-9965.EPI-14-0774 25378366

[b0080] Clarke N., McDevitt J., Kearney P.M., Sharp L. (2014). Increasing late stage colorectal cancer and rectal cancer mortality demonstrates the need for screening: a population based study in Ireland, 1994–2010. BMC Gastroenterol..

[b0085] Clarke N., McNamara D., Kearney P.M., O'Morain C.A., Shearer N., Sharp L. (2016). The role of area-level deprivation and gender in participation in population-based faecal immunochemical test (FIT) colorectal cancer screening. Prev. Med..

[b0090] Cochrane. PROGRESS-Plus: Cochrane Methods Equity [Available from: https://methods.cochrane.org/equity/projects/evidence-equity/progress-plus.

[b0095] Codd M.B., Laird O.M., Dowling M., Dervan P.A., Gorey T.F., Stack J.P., O'Herlihy B., Ennis J.T. (1994). Screening for breast cancer in Ireland: the Eccles Breast Screening Programme. Eur. J. Cancer Prev..

[b0105] Connolly S., Whyte R. (2019). Uptake of cancer screening services among middle and older ages in Ireland: the role of healthcare eligibility. Public Health.

[b0110] Coughlin S.S. (2019). Social determinants of breast cancer risk, stage, and survival. Breast Cancer Res. Treat..

[b0115] Coughlin S.S. (2020). Social determinants of colorectal cancer risk, stage, and survival: a systematic review. Int. J. Colorectal Dis..

[b0120] Cullerton K., Gallegos D., Ashley E., Do H., Voloschenko A., Fleming M., Ramsey R., Gould T. (2016). Cancer screening education: can it change knowledge and attitudes among culturally and linguistically diverse communities in Queensland, Australia?. Health Promot. J. Austr..

[b0125] Dahlgren G., Whitehead M. (2021). The Dahlgren-Whitehead model of health determinants: 30 years on and still chasing rainbows. Public Health.

[b0130] De Prez V., Jolidon V., Willems B., Cullati S., Burton-Jeangros C., Bracke P. (2021). Cervical cancer screening programs and their context-dependent effect on inequalities in screening uptake: a dynamic interplay between public health policy and welfare state redistribution. Int. J. Equity Health.

[b0135] De Prez V., Jolidon V., Cullati S., Burton-Jeangros C., Bracke P. (2022). Cervical cancer (over-)screening in Europe: Balancing organised and opportunistic programmes. Scand J Public Health.

[b0140] Department of Health. Department of Health. 2022. National Traveller Health Action Plan (NTHAP) 2022-2027. Available from: https://www.hse.ie/eng/services/publications/socialinclusion/national-traveller-health-action-plan-2022-2027.pdf.

[b0145] Department of Housing Local Government and Heritage (2016).

[b0150] Dietrich A.J., Tobin J.N., Cassells A., Robinson C.M., Greene M.A., Sox C.H., Beach M.L., DuHamel K.N., Younge R.G. (2006). Telephone care management to improve cancer screening among low-income women: a randomized, controlled trial. Ann. Intern. Med..

[b0155] Donnelly, D.W., Hegarty, A., Sharp, L., Carsin, A.-E., Deady, S., McCluskey, N., Comber, H., Gavin, A., 2013. The impact of adjustment for socioeconomic status on comparisons of cancer incidence between two European countries. J. Cancer Epidemiol. 2013:612514. DOI:10.1155/2013/612514. 10.1155/2013/612514 PMC3881585 24454373

[b0160] Duffy, K., Connolly, S., Nolan, A., Maître, B. 2022. Unequal chances? Inequalities in mortality in Ireland. Research Series.

[b0165] Dugord C., Franc C. (2022). Trajectories and individual determinants of regular cancer screening use over a long period based on data from the French E3N cohort. Soc. Sci. Med..

[b0170] Dunn S.F., Lofters A.K., Ginsburg O.M., Meaney C.A., Ahmad F., Moravac M.C., Nguyen C.T.J., Arisz A.M. (2017). Cervical and breast cancer screening after CARES: a community program for immigrant and marginalized women. Am. J. Prev. Med..

[b0180] European Commission. ECIS - European Cancer Information System. Long term incidence and mortality 2025-2040. [Available from: https://ecis.jrc.ec.europa.eu/explorer.php?$0-4$1-All$4-1,2$3-0$6-0,85$5-2020,2040$7-7$21-0$2-All$CLongtermChart1_1$X0_-1-AE27$CLongtermChart1_2$X1_-1-AE27$CLongtermChart1_3$X2_-1-AE27$CLongtermChart1_4$X3_14-$X3_-1-AE27$CLongtermTable1_6$X4_-1-AE27.

[b0190] European Commission. 2022. EU Beating Cancer Plan. Available from: https://ec.europa.eu/health/system/files/2022-02/eu_cancer-plan_en_0.pdf.

[b0195] Fentiman I.S. (2018). Unmet needs of men with breast cancer. Eur. J. Surg. Oncol..

[b0200] Fitzpatrick P., Fleming P., O'Neill S., Kiernan D., Mooney T. (2011). False-positive mammographic screening: factors influencing re-attendance over a decade of screening. J. Med. Screen..

[b0205] Fleming, P., O'Neill, S., Owens, M., Mooney, T., Fitzpatrick, P., 2013. Intermittent attendance at breast cancer screening. J. Public Health Res. 2:e14. DOI:10.4081/jphr.2013.e14. 10.4081/jphr.2013.e14 PMC4147734 25170485

[b0210] Gallagher F., Gallagher J. (2010). A closer look at cervical smear uptake and results pre-and post-introduction of the national screening programme. Ir. Med. J..

[b0215] Haase, T., Pratschke, J., 2016. The Pobal Hp Deprivation Index for Small Areas in th e Republic of Ireland. Pobal, Dublin.

[b0220] Health Information and Quality Authority. 2011. Recommendations for Unique Health Identifiers for Healthcare Practitioners and Organisations. Summary report. Health Information and Quality Authority, George’s Court, George’s Lane, Dublin 7.

[b0225] Health Service Executive. 2013. eHealth Strategy for Ireland.

[b0230] Health Service Executive, 2015. Knowledge and Information Strategy – Delivering the Benefits of eHealth in Ireland. DOI:https://www.ehealthireland.ie/knowledge-information-plan/hse_knowledge_information_plan-pdf.pdf.

[b0235] Health Service Executive. 2023. About Social Inclusion [Available from: https://www.hse.ie/eng/about/who/primarycare/socialinclusion/.

[b0240] Hiscock R., Bauld L., Amos A., Fidler J.A., Munafò M. (2012). Socioeconomic status and smoking: a review. Ann. N. Y. Acad. Sci..

[b0245] Hong Q.N., Fàbregues S., Bartlett G., Boardman F., Cargo M., Dagenais P., Gagnon M.-P., Griffiths F., Nicolau B., O’Cathain A., Rousseau M.-C., Vedel I., Pluye P. (2018). The Mixed Methods Appraisal Tool (MMAT) version 2018 for information professionals and researchers. Educ. Inf..

[b0250] Houses of the Oireachtas Committee on the Future of Healthcare. 2017. Houses of the Oireachtas Committee on the Future of Healthcare Sláintecare Report.

[b0255] Kavanagh J., Oliver S., Lorenc T. (2008). Reflections on developing and using PROGRESS-Plus. Equity Update.

[b0260] Lalor A., Redmond R. (2009). Breast screening for post-menopausal women: Following a survey in Ireland, Ann Lalor and Richard Redmond report on challenges that exist in providing breast cancer screening services for women with learning disabilities living in long-term care facilities. Learn. Disabil. Pract..

[b0265] Le Bonniec A., Meade O., Fredrix M., Morrissey E., O'Carroll R.E., Murphy P.J., Murphy A.W., Mc Sharry J. (2023). Exploring non-participation in colorectal cancer screening: a systematic review of qualitative studies. Soc. Sci. Med..

[b0270] Marlow L.A.V., Waller J., Wardle J. (2015). Barriers to cervical cancer screening among ethnic minority women: a qualitative study. J. Family Plann. Reproductive Health Care.

[b0275] Marques P., Nunes M., Antunes M.D.L., Heleno B., Dias S. (2020). Factors associated with cervical cancer screening participation among migrant women in Europe: a scoping review. Int. J. Equity Health.

[b0280] Marta J., Christian von W., Jane W., Dorota J., Aleksandra L., Jo W. (2012). Cervical screening among migrant women: a qualitative study of Polish, Slovak and Romanian women in London, UK. J. Family Plann. Reproductive Health Care.

[b0285] McCarron, M., Burke, E., McGlinchey, E., McCausland, D., 2011. Growing Older with an Intellectual Disability in Ireland 2011; First Results from the The Intellectual Disability Supplement to The Irish Longitudinal Study on Ageing.

[b0290] McCowan C., McSkimming P., Papworth R., Kotzur M., McConnachie A., Macdonald S., Wyke S., Crighton E., Campbell C., Weller D., Steele R.J.C., Robb K.A. (2019). Comparing uptake across breast, cervical and bowel screening at an individual level: a retrospective cohort study. Br. J. Cancer.

[b0295] McDevitt J., Comber H., Walsh P.M. (2017). Colorectal cancer incidence and survival by sub-site and stage of diagnosis: a population-based study at the advent of national screening. Ir. J. Med. Sci..

[b0300] McNamara D., Qasim A., Lee N., Condon C., O’Morain C. (2011). Round one of the Adelaide and Meath Hospital/Trinity College Colorectal Cancer Screening Programme: programme report and analysis based on established international key performance indices. Ir. J. Med. Sci..

[b0305] McNamara D., Leen R., Seng-Lee C., Shearer N., Crotty P., Neary P., Walsh P., Boran G., O'Morain C. (2014). Sustained participation, colonoscopy uptake and adenoma detection rates over two rounds of the Tallaght-Trinity College colorectal cancer screening programme with the faecal immunological test. Eur. J. Gastroenterol. Hepatol..

[b0310] Mihor A., Tomsic S., Zagar T., Lokar K., Zadnik V. (2020). Socioeconomic inequalities in cancer incidence in Europe: a comprehensive review of population-based epidemiological studies. Radiol. Oncol..

[b0315] Moore, P., Scarlett, S., Nolan, A., 2017. Health Insurance, Healthcare Utilisation and Screening, in C McGarrigle, O Donoghue, S Scarlett & R Kenny (eds), Health and Wellbeing: Active Ageing for Older Adults in Ireland: Evidence from The Irish Longitudinal Study on Ageing., Trinity College Dublin, Ireland., pp. 48-74.

[b0320] National Adult Literacy Agency. 2020. Literacy Now. Contract No.: ISBN: 978-1-907171-35-2Available from: https://www.nala.ie/research/literacy-for-life/.

[b0330] National Cancer Registry Ireland. 2018a. Cancer Factsheet Cervix. Available from: https://www.ncri.ie/sites/ncri/files/factsheets/Factsheet%20cervix.pdf.

[b0335] National Cancer Registry Ireland. 2018b. Cancer Factsheet Colorectal. Available from: https://www.ncri.ie/sites/ncri/files/factsheets/Factsheet%20colorectal.pdf.

[b0340] National Cancer Registry Ireland. 2022a. Breast, cervical and colorectal cancer 1994-2019: National trends for cancers with population-based screening programmes in Ireland. Available from: https://www.ncri.ie/sites/ncri/files/pubs/Trendsreport_breast_cervical_colorectal_22092022.pdf.

[b0345] National Cancer Registry Ireland. 2022b. Cancer Factsheet Breast. Available from: https://www.ncri.ie/sites/ncri/files/factsheets/Factsheet%20Female%20breast_corrected.pdf.

[b0355] National Screening Service. 2020a. BowelScreen Programme Report 2018 – 2019 Round Three. Health Service Executive, Available from: https://www.screeningservice.ie/publications/BowelScreen-Programme-Report-Round-Three.pdf.

[b0360] National Screening Service. 2020b. BreastCheck Programme Report 2018 and 2019. Health Servcies Executive, Available from: https://www.screeningservice.ie/publications/BreastCheck-Programme-Report_2018_and_2019.pdf.

[b0365] National Screening Service. 2021. LGBT+ Cervical Screening Study.: Health Service Executive. Available from: https://www.screeningservice.ie/publications/LGBT+Cervical-Screening-Study-Report.pdf.

[b0370] National Screening Service. 2022. CervicalCheck Programme Report 2017-2020. . Health Service Executive. Available from: https://www.screeningservice.ie/publications/CervicalCheck-ProgrammeReport-September-2017-March-2020.pdf.

[b0375] National Social Inclusion Office. Health Service Executive. 2018. Second National Intercultural Health Strategy 2018-2023.

[b0380] Ní Cheallaigh, C., Cullivan, S., Sears, J., Lawlee, A.M., Browne, J., Kieran, J., Segurado, R., O’Carroll, A., O’Reilly, F., et al., 2017. Usage of unscheduled hospital care by homeless individuals in Dublin, Ireland: a cross-sectional study. BMJ Open 7:e016420. DOI:10.1136/bmjopen-2017-016420. 10.1136/bmjopen-2017-016420 PMC5719262 29196477

[b0385] Ní Riain A., Stewart M., Phelan D., Bury G., Mulcahy F. (2001). Cervical smears: comparison of knowledge and practice of a general practice sample with a high-risk group. Int. J. STD AIDS.

[b0390] O'Brien K.M., Mooney T., Fitzpatrick P., Sharp L. (2018). Screening status, tumour subtype, and breast cancer survival: a national population-based analysis. Breast Cancer Res. Treat..

[b0395] O'Brien K.M., Sharp L. (2013). Trends in incidence of, and mortality from, cervical lesions in Ireland: baseline data for future evaluation of the national cervical screening programme. Cancer Epidemiol..

[b0400] O'Donovan B., Mooney T., Rimmer B., Fitzpatrick P., Flannelly G., Doherty L., Martin C., O'Leary J., O'Connor M., Sharp L. (2021). Advancing understanding of influences on cervical screening (non)-participation among younger and older women: a qualitative study using the theoretical domains framework and the COM-B model. Health Expect..

[b0405] O'Neill J., Tabish H., Welch V., Petticrew M., Pottie K., Clarke M., Evans T., Pardo Pardo J., Waters E., White H., Tugwell P. (2014). Applying an equity lens to interventions: using PROGRESS ensures consideration of socially stratifying factors to illuminate inequities in health. J. Clin. Epidemiol..

[b0410] Pavee Point Traveller and Roma Centre & Mid West Traveller Health Unit. 2019. Traveller Health Needs Assessment: COUNTY CLARE. Mid West Community Healthcare Traveller Health Unit, Limerick. Contract No.: IBSN 1897598416Available from: https://www.paveepoint.ie/wp-content/uploads/2019/07/Clare-Needs-Assessment.pdf.

[b0415] Policy Drugs Unit Social Inclusion. Department of Health. 2020. Reducing Harm, Supporting Recovery Progress Report 2020.

[b0420] Raffle A.E., Mackie A., Gray J.A.M. (2019).

[b0425] Rollet Q., Tron L., De Mil R., Launoy G., Guillaume É. (2021). Contextual factors associated with cancer screening uptake: a systematic review of observational studies. Prev. Med..

[b0430] Sharp L., Donnelly D., Hegarty A., Carsin A.E., Deady S., McCluskey N., Gavin A., Comber H. (2014). Risk of several cancers is higher in urban areas after adjusting for socioeconomic status. Results from a two-country population-based study of 18 common cancers. J. Urban Health.

[b0435] Smith D., Thomson K., Bambra C., Todd A. (2019). The breast cancer paradox: a systematic review of the association between area-level deprivation and breast cancer screening uptake in Europe. Cancer Epidemiol..

[b0440] Tatari C.R., Andersen B., Brogaard T., Badre-Esfahani S.K., Jaafar N., Kirkegaard P. (2020). Perceptions about cancer and barriers towards cancer screening among ethnic minority women in a deprived area in Denmark – a qualitative study. BMC Public Health.

[b0445] The Department of Justice and Equality. The Department of Justice and Equality. 2019. National LGBTI+ Inclusion Strategy 2019-2021.

[b0450] Tulchinsky, T.H., Varavikova, E.A., 2014. Measuring, Monitoring, and Evaluating the Health of a Population. The New Public Health:91-147. DOI:10.1016/b978-0-12-415766-8.00003-3.

[b0455] Tyndall, J. 2010. The AACODS Checklist is Designed to Enable Evaluation and Critical Appraisal of Grey Literature Flinders University [Available from: https://www.library.sydney.edu.au/research/systematic-review/downloads/AACODS_Checklist.pdf.

[b0460] Ullah M.F., Fleming C.A., Mealy K. (2018). Changing trends in age and stage of colorectal cancer presentation in Ireland - From the nineties to noughties and beyond. Surgeon.

[b0465] Walji L.T., Murchie P., Lip G., Speirs V., Iversen L. (2021). Exploring the influence of rural residence on uptake of organized cancer screening - a systematic review of international literature. Cancer Epidemiol..

[b0470] Walsh, B., Domhnaill, M., Mohan, G., 2021. Developments in healthcare information systems in Ireland and internationally. ESRI. 'Available from:https://www.esri.ie/publications/developments-in-healthcare-information-systems-in-ireland-and-internationally.

[b0475] Walsh P.M., McDevitt J., Deady S., O’Brien K., Comber H. (2016).

[b0480] Walsh B., Silles M., O'Neill C. (2010). A cross-border comparison of breast and cervical cancer screening uptake. Ir. Med. J..

[b0485] Walsh B., Silles M., O'Neill C. (2012). The role of private medical insurance in socio-economic inequalities in cancer screening uptake in Ireland. Health Econ..

[b0490] World Health Organisation. 2017. National health inequality monitoring: a step-by-step manual. . Geneva Report No.: Licence: CC BY-NC-SA 3.0 IGO. Available from: https://cdn.who.int/media/docs/default-source/gho-documents/9-june-web-version-17136-national-health-inequality-monitoring-step-by-step-manual.pdf?sfvrsn=2a9ac9a9_2.

[b0495] World Health Organisation. 2020. All Cancers Factsheet. Source: Globocan 2020: The Global Cancer Observatory. https://gco.iarc.fr/. [13th Feb 2022]. Available from: https://gco.iarc.fr/today/data/factsheets/cancers/39-All-cancers-fact-sheet.pdf.

[b0500] World Health Organisation. 2022. A short guide to cancer screening. Increase effectiveness, maximize benefits and minimize harm. Copenhagen: WHO Regional Office for Europe Available from: https://apps.who.int/iris/bitstream/handle/10665/351396/9789289057561-eng.pdf.

