# Supplementary material for: How is equity captured for colorectal, breast and cervical cancer incidence and screening in the Republic of Ireland: A review
Source: Prev Med Rep. 2023 Sep 17;36:102405. doi: 10.1016/j.pmedr.2023.102405 (PMC10518567; doi:10.1016/j.pmedr.2023.102405)
Supplement: Supplementary material 2 [file mmc2.docx]

**Cancer screening equity review Ireland- Supplementary files**

1. **PubMed search strategy keywords**

**Keywords for Ireland**

1. (Ireland OR Irish)[all fields]

**Keywords for incidence rates**

1. Incidence[MeSH] OR Risk[MeSH]
2. ((incidence[Title/Abstract]) OR (survival[Title/Abstract])) OR (mortality[Title/Abstract])
3. #1 OR #2

**Keywords for breast cancer**

1. (breast cancer[MeSH Terms]) OR (breast dysplasia[MeSH Terms])
2. breast cancer*[Title/Abstract] OR breast carcinoma*[Title/Abstract] OR breast tumo*[Title/Abstract] OR breast neoplasm*

**Keywords for cervical cancer**

1. ((cervical cancer, uterine[MeSH Terms]) OR (cervical dysplasia, uterine[MeSH Terms])) OR (cervical intraepithelial neoplasia[MeSH Terms])
2. ((((cervical cancer[Title/Abstract]) OR (cervical neoplasm[Title/Abstract])) OR (cervical tumour[Title/Abstract])) OR (cervical tomor[Title/Abstract])) OR (cervical carcinoma[Title/Abstract])

**Keywords for colorectal cancer**

1. ((cancer, colorectal[MeSH Terms]) OR (cancer, colonic[MeSH Terms])) OR (rectal cancer[MeSH Terms])
2. ((colorectal*[Title/Abstract] OR CRC[Title/Abstract] OR colon*[Title/Abstract] OR bowel*[Title/Abstract] OR rectal[Title/Abstract] OR rectum[Title/Abstract] OR sigmoid[Title/Abstract] OR anal[Title/Abstract] OR anus) n3 (cancer[Title/Abstract] OR neoplasm*[Title/Abstract] OR tumor*[Title/Abstract] OR tumour[Title/Abstract] OR carcinom*[Title/Abstract] OR sarcom*[Title/Abstract] OR adenocarcinom*[Title/Abstract] OR adeno?carcinom*[Title/Abstract] OR adenom*[Title/Abstract] OR lesion*[Title/Abstract]))

**Keywords for participation rates**

1. Patient Participation [MeSH] OR Patient compliance[MeSH]
2. (uptake OR takeup OR attend* OR accept* OR adhere* OR participat* OR utilisat* OR utilizat* OR engage* OR promot* OR consent* OR complie* OR comply OR compliance).ti,ab.
3. (noncompliance OR non compliance OR reluctan* OR nonrespon* OR non respon* OR non-attend* OR non attend* OR dropout OR drop out).ti,ab.
4. #1 OR #2 OR #3

**Keywords for cervical cancer screening**

1. papanicolaou smear[MeSH Terms] OR Vaginal Smears/ OR cervical smear[MeSH Terms]
2. cancer, uterine cervical[MeSH Terms] OR cervical dysplasia, uterine[MeSH Terms] OR cervical intraepithelial neoplasia[MeSH Terms]
3. Mass Screening[MeSH Terms]
4. #2 AND #3
5. ((pap OR hpv OR human papilloma virus OR cervi* OR vagina* OR cytology) n3 (test* OR smear* OR screen* OR swab*)).ti,ab.
6. #1 OR #4 S7 OR #5

**Keywords for breast cancer screening**

1. mammography[MeSH]
2. breast cancer[MeSH] OR breast dysplasia[MeSH]
3. mass screening [MeSH] OR Early Detection of Cancer[MeSH]
4. #2 AND #3
5. ((breast cancer* OR breast carcinoma* OR breast tumo* OR breast neoplasm*) n3 (test* OR screen* OR mammogra*)).ti,ab.
6. #1 OR #4
7. #5 OR #6

**Keywords for bowel cancer screening**

1. blood, occult[MeSH Terms] OR immunochemistry[MeSH Terms]
2. cancer, colorectal[MeSH Terms] OR colonic cancer[MeSH Terms] OR rectal cancer[MeSH Terms]
3. mass screening [MeSH]
4. #1 AND #2
5. #2 AND #3
6. #4 OR #5
7. ((colorectal* OR CRC OR colon* OR bowel* OR  rectal OR rectum OR sigmoid OR anal OR anus) n3 (cancer OR neoplasm* OR tumor* OR tumour OR carcinom* OR sarcom* OR adenocarcinom* OR adeno?carcinom* OR adenom* OR lesion*)).ti,ab.
8. (screen* OR test* OR (population* n2 surveillance) OR (early n3 detect*) OR (early n3 prevent*)).ti,ab.
9. #7 AND #8
10. ((faecal OR fecal OR feces OR faeces OR gFOBT OR FOBT OR FOB OR FIT OR haemoccult OR hemoccult OR occult blood OR (stool n3 occult)) n3 (test* OR screen* OR diagn*)). ti,ab.
11. (immunochemical* n3 (test* OR screen* OR diagn*)) OR (immunologic* n3 (test* OR screen* OR diagn*)).mp.
12. #10 OR #11
13. #6 OR #9 OR #12
14. **Data extraction tool**

| **Title** | **DOI/URL** | **Literature type** | **Incidence/ participation** | **Population** | **Aims** | **Methods** | **Condition** | **Equity measures** | **Summary of findings** |
| --- | --- | --- | --- | --- | --- | --- | --- | --- | --- |
| Title of study/ report | Link to study/ report | Peer-reviewed/ grey literature | Incidence of cancer/ participation of screening literature | Describe the population studied | Describe the aims | Describe the methods | List Breast/ cervical/ colorectal cancer | List what PROGRESS-Plus stratifiers were used | Summarise variation/no variation in outcome by equity stratifier |
